# Case of Spinal Neuralgia

**Published:** 1829-01-01

**Authors:** 


### LIII.

### CASE OF SPINAL NEURALGIA.

Morbid anatomy is an excellent thing, but it sometimes leads its followers, like an ignis fatuus, into the mud! Thus, some may be found at the present day, (we trust, for the sake of their patients if any such unfortunates belong to them, that their number is but small,) who sneer at the term irritation, and see but inflammation in every malady and every pain. We cannot conceive a greater pest let loose upon the public in the shape of a medical practitioner, than a man bred up with any such ideas. He will do more harm with his purges and his lancet than the savage Attila, who boasted that the grass never grew where his horse had trod! Of late years much has been done towards promoting the knowledge we possess of the neuralgiæ, but much, very much, remains behind. Dr. Macculloch, by his able and elaborate work, has fully and fairly earned golden opinions, and we trust that his labours will have a most salutary effect on the minds of the profession. Another gentleman, Mr. Brodie, pursuing a totally different walk, has paid much attention to neuralgic and hysterical affections, especially those which attack the external parts. He has stated, and to this we have already alluded, that nine out of ten of those who have been doctored for spinal complaints, have had in reality none at all, the whole being nothing but nervous or hysterical pains. A good illustration of this sort of affection is related by Mr. Allan, in the Glasgow Medical Journal.

"Mary Durand, a single woman, 24 years of age, applied to me on the 25th of June last for relief, complaining of pain in the left side, under the mamma, not in the gland. The pain was much aggravated by pressing with the fingers against the ribs, and sometimes, she said, it extended down the side, as far as the crest of the ilium. It was accompanied with pain at the top and towards the back part of the left shoulder, which she described as a sensation of burning, not interfering with the movements of the joint, but so tender to the touch was the part, that she could not rest on that side in bed, and always awoke in severe pain, if she happened to turn upon it. She had not been altogether free from either of those pains for nearly four years, but they had always been worse for a few months every spring and autumn. About seven years ago she had been suddenly attacked, while kneeling, with severe pain in the left knee, darting up the thigh and into the loins. Considerable swelling and local inflammation had then appeared in the knee, but were soon removed, while the pain in the knee and loins lasted, with little or no intermission, for three years and a half, when, while walking, she was surprised to find her lameness suddenly leave her, and, at the same instant, the pains in her side and shoulder came on for the first time.

"Her complexion was pale and sallow, with an aspect expressive of habitual suffering. Her tongue was slightly furred and moist; pulse feeble. She was emaciated, and had no relish for food; bowels inclined to costive. She had begun to menstruate very soon after 12 years of age, but the discharge had never occurred oftener than once in five or six weeks. The discharge was copious, unaccompanied with uterine pain, and generally lasted for eight days. About two years ago, however, it had become less in quantity, of a paler colour, and accompanied with pain, though still lasting eight days.

"Having perused Dr. Brown's paper on Irritation of the Spinal Nerves, shortly before this patient presented herself, I was led to examine her spine, when I found that there were three or four of the middle dorsal vertebræ, the most moderate pressure upon which gave her considerable pain in the part, and increased that in her side. She had never previously felt pain in that part of her back, although the tenderness was too unequivocal, and too distinctly limited in extent, to leave any doubt as to its reality."

A few leeches were applied to the spine, which immediately relieved the pain in the side. A drachm of the carbonate of iron was then given three times a day, which improved the appearance and relieved the pain, and limited its appearance to every evening. The pulvis cinchonæ and carbonate of soda were exhibited every four hours instead of the carbonate of iron, and a blister applied to the spine. The pain was still more relieved. When the blister was healed, tartar emetic ointment was rubbed upon the spine, and half an ounce of castor oil with the same amount of the oleum terebinthinæ, given on the 14th of July, to relieve constipation and promote the menstrual discharge. At bed-time this was followed by a purgative of calomel and jalap, which brought away a quantity of highly offensive bottle-green fæces.

"She was now directed to take half a drachm of oleum terebinthinæ, mixed with five minims of liquor potassæ, and half an ounce of cinnamon water, three times a day. The jalap and calomel were likewise ordered to be taken every second or third night. This practice being continued till the 4th of August, an enormous quantity of scybalous and green fæces, with four large lumbrici, and a vast number of ascarides, were discharged. The turpentine mixture was then ordered to be still continued, but instead of the jalap and calomel powders, a pill containing four grains of the compound colocynth pill, with half a grain of calomel, was directed to be taken every night and morning. This plan was pursued till the 18th of August, when the pains had completely disappeared, her alvine evacuations had become healthy, her complexion clear, her appetite good, and her nights passed in sound refreshing sleep on either side."

The above was, beyond all doubt, a case of hysteric or neuralgic pains, call it which you will, occurring, as such very frequently do, in an unmarried female, labouring under irregular menstrual discharge. A young woman is suddenly affected with pain in the knee, which lasts for three years and a half, and as suddenly leaves her, giving place to another painful affection, extending from the shoulder to the crest of the ilium. Is such the history of actual organic disease? The answer is obvious enough. The character of the pain was precisely that presented by the neuralgiæ—it was aggravated by even very moderate pressure. Nothing can be more characteristic than this—the extravagant extent of the surface on which the pain is spread, and its production or aggravation by the slightest touch. The treatment we highly approve of, save that we are inclined to express our doubts as to whether the relief from the blistering and leeching to the spine was not a coincidence rather than a consequence: of this we are sure, that similar cases repeatedly do well without the application of a blister or a leech, the spine of the patient being just as unaffected as our's at the present time of writing. One word with regard to the oleum terebinthinæ. We believe it to be a very valuable remedy in cases of deficient menstruation, attended (they commonly go hand in hand) with a costive state of bowels.

